# Supplementary material for: Cryo electron tomography with volta phase plate reveals novel structural foundations of the 96-nm axonemal repeat in the pathogen Trypanosoma brucei
Source: eLife. 2019 Nov 11;8:e52058. doi: 10.7554/eLife.52058 (PMC6974359; doi:10.7554/eLife.52058)
Supplement: Supplementary file 1. [file elife-52058-supp1.pptx]

## Slide 1
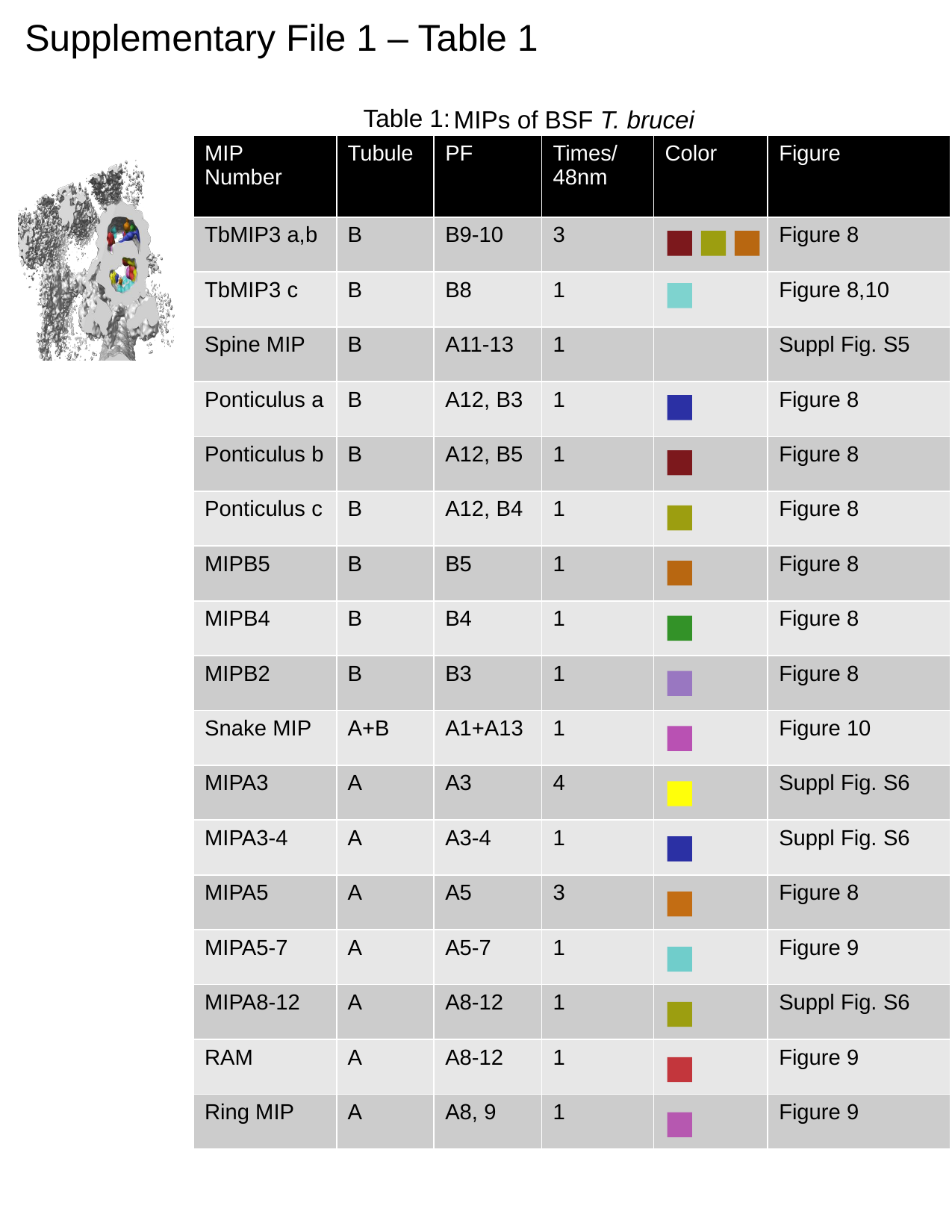

Supplementary File 1 – Table 1
Table 1:
MIPs of BSF T. brucei
| MIP Number | Tubule | PF | Times/48nm | Color | Figure |
| --- | --- | --- | --- | --- | --- |
| TbMIP3 a,b | B | B9-10 | 3 | | Figure 8 |
| TbMIP3 c | B | B8 | 1 | | Figure 8,10 |
| Spine MIP | B | A11-13 | 1 | | Suppl Fig. S5 |
| Ponticulus a | B | A12, B3 | 1 | | Figure 8 |
| Ponticulus b | B | A12, B5 | 1 | | Figure 8 |
| Ponticulus c | B | A12, B4 | 1 | | Figure 8 |
| MIPB5 | B | B5 | 1 | | Figure 8 |
| MIPB4 | B | B4 | 1 | | Figure 8 |
| MIPB2 | B | B3 | 1 | | Figure 8 |
| Snake MIP | A+B | A1+A13 | 1 | | Figure 10 |
| MIPA3 | A | A3 | 4 | | Suppl Fig. S6 |
| MIPA3-4 | A | A3-4 | 1 | | Suppl Fig. S6 |
| MIPA5 | A | A5 | 3 | | Figure 8 |
| MIPA5-7 | A | A5-7 | 1 | | Figure 9 |
| MIPA8-12 | A | A8-12 | 1 | | Suppl Fig. S6 |
| RAM | A | A8-12 | 1 | | Figure 9 |
| Ring MIP | A | A8, 9 | 1 | | Figure 9 |
